# After-War Reorganisation

**Published:** 1919-09-13

**Authors:** 


### THE LONDON HOSPITAL.

### After-War Reorganisation.

THE quarterly report of the House Committee for the third quarter of the year 1919, which was presented at the Court of September 3, records that considerable progress has been made towards reorganising the work of the hospital after the disorganisation inevitably caused by the war. The adequate staffing of the hospital with doctors and nurses in the past had been a constant source of difficulty. The medical staff nominally consists of thirty-six honorary physicians and surgeons, twenty-eight resident doctors—house-physicians and house-surgeons—and eleven non-resident qualified men, besides many others engaged in laboratory work. During the war the majority of the honorary staff were engaged in the medical service of either the Army or Navy, while the work of the resident doctors was carried out by a diminished staff, consisting of men who were, owing to some physical disability, unable to serve, and by lady doctors. These residents were paid for their services. The work of the hospital generally had to be curtailed.

### WORK (?) IN THE PACIFIC.

Now the members of the honorary staff have resumed hospital duty, and the only difficulties are as regards filling the resident and non-resident junior appointments. These difficulties are quickly disappearing, and an adequate number of applications for the posts, which are now for the most part honorary, is being received from demobilised doctors. The majority of applications are from old "Londoners," who wish to return to the hospital to rub up their medical and surgical knowledge as house-physicians and house-surgeons under our experienced staff. Though during the war some gained useful experience, there was little opportunity for the exercise of the skill and knowledge required for ordinary civilian practice. One doctor, for instance, as soon as he was qualified, joined the Medical Department of the Navy, was sent to an island in the Pacific, where he remained for three and a-half years, during which time almost the only case he was called upon to deal with was that of a man who had broken his neck.

### SHORTAGE OF PROBATIONERS.

In regard to the nursing staff the prospect is improving. In the early days of the war—in fact, for the first two years—there was a rush of candidates as paying probationers, who were desirous of gaining sufficient knowledge in order to fit them for naval or military nursing. Subsequently there was considerable shortage owing to women of the class from which nurses are drawn having become settled in some war work. This shortage is still continuing. For some considerable time after the Armistice the number of applications was disappointing. The cause of this is probably to be found in the fact that many women, after the stress of war work, considered that they were entitled to a spell of leisure and a good time before taking any settled occupation. For keeping up the succession of nurses thirty-two probationers are required for each course of seven weeks' preliminary training at Tredegar House. The number of applicants has just shown a satisfactory increase, but more candidates are still needed.

### RETIREMENT OF SISTER CURRIE.

Miss Beatley (Sister Currie) has retired after thirty-four years' service in the hospital. It was her intention to have retired earlier, but, like others of the nursing staff, she stayed on to help tide over the difficult period of the war. In addition to Currie Ward, Miss Beatley had charge of the resident doctors when ill, and many generations of housemen have cause to be grateful for her skill and care.

### AN ACCUMULATION OF CASES.

The demands in regard to patients are no less than in pre-war days; in fact, there is an accumulation of non-urgent cases.

During the war a person suffering from some complaint which was not urgent—hernia, for instance—unless it was a question of operation to fit him for active service, had to be told that he could not be attended to till after the war. It is of cases of this type that there is a considerable accumulation. It is noticeable that many women patients, owing to being in good work during the war, have managed to carry on and put off coming to hospital for treatment.

At the present time there are no fewer than 681 patients awaiting admission.

### INCREASING EXPENSES.

Turning to finance, the situation is not without anxieties. The quinquennial appeal last year was a success, but increase in income is by no means proportionate to the rise in prices. The expenses of the hospital have gone up enormously—£205,000 in 1918, as compared with £119,000 in 1913. The milk bill alone will this year be about £14,000. At the present moment there is a loan from the hospital's bankers of £19,000.

### CONVALESCENT HOME AT REIGATE.

The executors of the late Mr. W. C. Alexander have given to the hospital a Convalescent Home at Reigate. This Home during the war was used for soldiers, and in giving it to the hospital the executors have carried out the late Mr. Alexander's wishes that the buildings should still be used for convalescent work. The Home is conveniently situated, twenty-one miles from London, and contains thirty beds.

### RESIGNATIONS.

The resignation was announced of Sir William Lister, K.C.M.G., from the post of ophthalmic surgeon, and that of Dr. Lambert Lack from the post of aural surgeon.

